# Diagnostic yield and clinical relevance of expanded germline genetic testing for nearly 7000 suspected HBOC patients

**DOI:** 10.1038/s41431-023-01380-2

**Published:** 2023-05-15

**Authors:** Jan Henkel, Andreas Laner, Melanie Locher, Tobias Wohlfrom, Birgit Neitzel, Kerstin Becker, Teresa Neuhann, Angela Abicht, Verena Steinke-Lange, Elke Holinski-Feder

**Affiliations:** 1 MGZ – Medizinisch Genetisches Zentrum, München, Germany; 2 Friedrich-Baur-Institute, Department of Neurology, Klinikum der Universität, Ludwig-Maximilians-Universität, München, Germany; 3 Medizinische Klinik und Poliklinik IV, Campus Innenstadt, Klinikum der Universität, München, Germany

**Keywords:** Breast cancer, Genetic testing

## Abstract

Here we report the results of a retrospective germline analysis of 6941 individuals fulfilling the criteria necessary for genetic testing of hereditary breast- and ovarian cancer (HBOC) according to the German S3 or AGO Guidelines. Genetic testing was performed by next-generation sequencing using 123 cancer-associated genes based on the Illumina TruSight® Cancer Sequencing Panel. In 1431 of 6941 cases (20.6%) at least one variant was reported (ACMG/AMP classes 3–5). Of those 56.3% (*n* = 806) were class 4 or 5 and 43.7% (*n* = 625) were a class 3 (VUS). We defined a 14 gene HBOC core gene panel and compared this to a national and different internationally recommended gene panels (German Hereditary Breast and Ovarian Cancer Consortium HBOC Consortium, ClinGen expert Panel, Genomics England PanelsApp) in regard of diagnostic yield, revealing a diagnostic range of pathogenic variants (class 4/5) from 7.8 to 11.6% depending on the panel evaluated. With the 14 HBOC core gene panel having a diagnostic yield of pathogenic variants (class 4/5) of 10.8%. Additionally, 66 (1%) pathogenic variants (ACMG/AMP class 4 or 5) were found in genes outside the 14 HBOC core gene set (secondary findings) that would have been missed with the restriction to the analysis of HBOC genes. Furthermore, we evaluated a workflow for a periodic re-evaluation of variants of uncertain clinical significance (VUS) for the improvement of clinical validity of germline genetic testing.

## Introduction

About 20% of all familial cases of hereditary breast and ovarian cancer (HBOC; MIM: 114480, 167000) are caused by pathogenic heterozygous variants in the two major risk genes *BRCA1* (MIM: 113705) and *BRCA2* (MIM: 600185) [1, 2] and since breast cancer is one of the leading causes of death in women worldwide, the genes associated with breast cancer are certainly among the most analysed in humans. Over the past decades, other high- and moderate-penetrance genes besides *BRCA1* and *BRCA2* have been linked to HBOC [3, 4].

In general, predisposition to HBOC can be assigned to one of three classes: (1) rare but highly penetrant variants in the genes *BRCA1*, *BRCA2, PALB2* (MIM: 610355) and *TP53* (MIM: 191170); (2) rare, moderately penetrant variants, e.g. in the genes *CHEK2* (MIM: 604373) and *ATM* (MIM: 607585); and (3) variants with higher frequency in the population (single nucleotide variants [SNVs]), which individually represent only a very small increase in risk, but which can be combined to form a polygenic risk score (PRS) [5, 6]. Unfortunately, all known high, moderate, and low risk genes or alleles identified so far leave the majority of BC and OC cases unexplained [7] or, in ~5–20% of the cases, result in the detection of so-called variants of uncertain significance (VUS) at the time of reporting [8–10]. The identification of individuals carrying pathogenic variants in genes associated with HBOC is nevertheless very important, as this is the basis of personalised medical care for patients and at-risk family members [11–14].

This study provides data of 6941 suspected HBOC patients analysed for 123 genes based on the Illumina TruSight® Cancer Sequencing Panel with regard to positive findings (e.g. a variant was reported) including the grading of the corresponding variant according to ACMG/AMP (American College of Medical Genetics and Genomics, Association for Molecular Pathology) criteria [15].

Although there are recommendations at national and international level as to which genes should be analysed in the context of a hereditary breast and ovarian cancer diagnosis the individual composition of the corresponding gene panels varies greatly and there are still major differences in the diagnostic approach [16, 17] and consequently in the diagnostic yield (Supplementary Table S1). Despite these different panel strategies, studies have shown the clear clinical utility of multi-gene panels for HBOC testing [16, 18].

In Germany, the German HBOC Consortium has recommended analysing 11 genes as part of HBOC testing [19]; internationally, ClinGen (“Breast Cancer Gene Curation Expert Panel” = 11 genes and “Ovarian Cancer Gene Curation Expert Panel” = 11 genes) (https://www.clinicalgenome.org/affiliation/40042/) as well as the PanelApp from Genomics England (“Inherited breast cancer and ovarian cancer” = 5 genes, “Pertinent cancer susceptibility” = 5 genes and the “Familial breast cancer” = 26 genes) (https://panelapp.genomicsengland.co.uk/) recommend other genes, with *BRCA1*, *BRCA2* and *PALB2* being the only genes included in all recommendations. The situation is aggravated by the fact that the content of the different recommended gene panels for HBOC has been changed several times over the last decade due to new gene-disease associations. For the present work, we have structured the results in such a way that we can compare an internal 14-gene HBOC core panel with the alternative gene panels described above. In addition, we were able to further recognise other tumour risk syndromes (TRS) based on the underlying 123 gene backbone. Furthermore, we present data for the first 106 re-evaluated VUSs revaluated on a regular basis, significantly improving variant classification [20–24].

## Methods

### Subjects

A total of 6941 patients fulfilling the criteria necessary for genetic testing in Germany (S3 [19] or AGO Guidelines https://www.ago-online.de/ago-kommissionen/kommission-mamma) gave informed consent for genetic testing with regard to HBOC including secondary findings.

### Study design

We retrospectively analysed our cohort which had been tested by Next-Generation Sequencing (NGS; Illumina NextSeq and NovaSeq) according to diagnostic yield (ACMG/AMP classes 3, 4 and 5) and secondary findings. We defined the core HBOC gene panel with the following 14 genes: *BRCA1*, *BRCA2*, *ATM*, *CDH1*, *CHEK2*, *NF1*, *PALB2*, *PTEN*, *RAD51C*, *RAD51D*, *STK11*, *TP53*, *BARD1* and *BRIP1* and used an Agilent SureSelectXT gene panel custom kit containing 123 genes (“Hereditary Cancer Syndromes—Comprehensive Panel”) as an enrichment backbone. The 123 gene set is based on the Illumina TruSight® Cancer Sequencing Panel (Cat. No. C-121-0202), a detailed list of the covered 123 genes can be found in Supplementary Table S1.

In total, 4867 datasets were analysed for copy number variants (CNV´s) using MLPA (MRC Holland; SALSA MLPA BRCA1 P002 and BRCA2 P045) technologies, for all other cases the CNV analysis was performed using NGS.

### High throughput sequencing and bioinformatics pipeline

DNA samples were extracted from peripheral blood (2–4 ml EDTA). Gene targeted enrichment was performed with the SureSelectXT gene panel custom kit (Agilent Biosciences) or the Twist Human Comprehensive Exome Kit (Twist Biosciences). Massively parallel sequencing was carried out on an Illumina NextSeq 500 or a Novaseq 6000 system (Illumina, San Diego, CA) as 150 bp paired-end runs using v2.0 SBS chemistry. Sequencing reads were aligned to the human reference genome (GRCh37/hg19) using BWA (v0.7. 13-r1126) with standard parameters. Statistics on coverage and sequencing depth on the clinical targeted regions (i.e. RefSeq coding exons and ±5 intronic region) were calculated with a custom script.

Gene panel enrichment-based SNV and INDEL calling was conducted using SAMtools (v1.3.1) with subsequent coverage and quality dependent filter steps. Variant annotation was performed with snpEff (v4.2) and Alamut-Batch (v1.4.4). CNV calling was performed using is a combination of four open source tools (ExomeDepth, Clamms, Canoes, Codex) and an in-house developed method (Mann–Whitney *U* test and heterozygosity check). A call was considered if at least two out of the five methods are concordant for the respective CNV.

Exome enrichment-based SNV, INDEL and CNV calling was conducted with VARFEED worker 1.5.1 (Varvis, Limbus Technologies). Only SNVs and small INDELs in the coding and flanking intronic regions (±50 bp) were evaluated.

### Classification of genetic variants

The variant interpretation was done by certified molecular and clinical geneticists specialised in data analysis and annotation with significant relevant expertise. Data from multiple sources were evaluated for the assessment of the potential pathogenicity of each variant. The variants were classified according to the ACMG/AMP guidelines with the 5-tier classification system: class 5 (pathogenic), class 4 (likely pathogenic), class 3 (variants of unknown significance, VUS), class 2 (likely benign) and class 1 (benign) [15]. The primary literature cited by HGMD curators, PubMed and Mastermind Genomic Search Engine (https://www.genomenon.com/mastermind) was reviewed. Variant interpretation was compared to the most established variation reference databases (HGMD and ClinVar). HGMD represents an attempt to collate all known (published) gene lesions responsible for human inherited disease [25]. ClinVar is a freely accessible, public archive of reports of the relationship among human variations and phenotypes, with supporting evidence [26]. ClinVar uses standard terms for clinical significance recommended by an authoritative source when available. These standards include: the 5-tier classification system for Mendelian diseases recommended by ACMG/AMP. Differences in interpretation among submitters within those five tiers are reported as a conflict using the phrase “conflicting interpretations of pathogenicity”.

Generally we submit all variants we list in diagnostic reports to the LOVD [https://www.lovd.nl/3.0/home] and/or ClinVar [26].

### Periodic re-evaluation of variants of uncertain significance

We have implemented a recall system in our LIMS that marks patient findings with VUS in a 2-year cycle. This system was launched in 2018 and the first VUS were re-evaluated from 2020 (*n* = 106). For the re-evaluation, a thorough database search was performed and the variants were (re-)classified according to the latest recommendations of the “Sequence Variant Interpretation Working Group“ (SVI) (https://clinicalgenome.org/working-groups/sequence-variant-interpretation/) of the ACMG/AMP classification guidelines [15].

## Results

Of the 6941 individuals at least one variant of ACMG/AMP class 3 to 5 was reported in 1431 cases (20.6%) (Supplementary Table S1) based on the comprehensive 123 gene panel (“Hereditary Cancer Syndromes—Comprehensive Panel”). Of these 1431 cases, 806 (56.3%) carry at least one ACMG/AMP class 4 or 5 variant (Table 1), while 625 (46.7%) only class 3 variant(s) (VUS) (Supplementary Table S2).

**Table 1:** Found pathogenic variants applying different gene panels.

Gene panel	# of genes	class 4/5	cases	Dx yield
HBOC core panel	14	759	747	10.8%
German HBOC Consortiums Panel	11	751	739	10.6%
ClinGen: Breast Cancer Gene Curation Expert Panel	11	733	722	10.4%
ClinGen: Ovarian Cancer Gene Curation Expert Panel	11	564	560	8.1%
Genomics England PanelApp: Inherited breast cancer and ovarian cancer	5	715	704	10.1%
Genomics England PanelApp: Pertinent cancer susceptibility	5	541	538	7.8%
Genomics England PanelApp: Familial breast cancer	26	795	778	11.2%
Hereditary Cancer Syndromes—Comprehensive Panel	123	825	806	11.6%

A comprehensive list of all pathogenic variants (ACMG/AMP class 4/5) within the various recommended HBOC gene panels. Given are all the potentially solved cases using the corresponding panel and the resulting diagnostic yield. See Supplementary Table S1 for further information regarding the genes covered by these panels.

If the above mentioned national and international gene panel recommendations were followed (German Consortium, ClinGen Expert Panel, Genomics England PanelsApp), differences in diagnostic yields for pathogenic variants (class 4/5) would range from 7.8 to 11.6% (Table 1). Considering the variants of uncertain significance as well, a variant (class 3–5) would have been reported in 11.5% (Genomics England PanelApp: Pertinent cancer susceptibility) to 20.6% (Hereditary Cancer Syndromes—Comprehensive Panel) of cases (Supplementary Table S1).

**Fig. 1:**
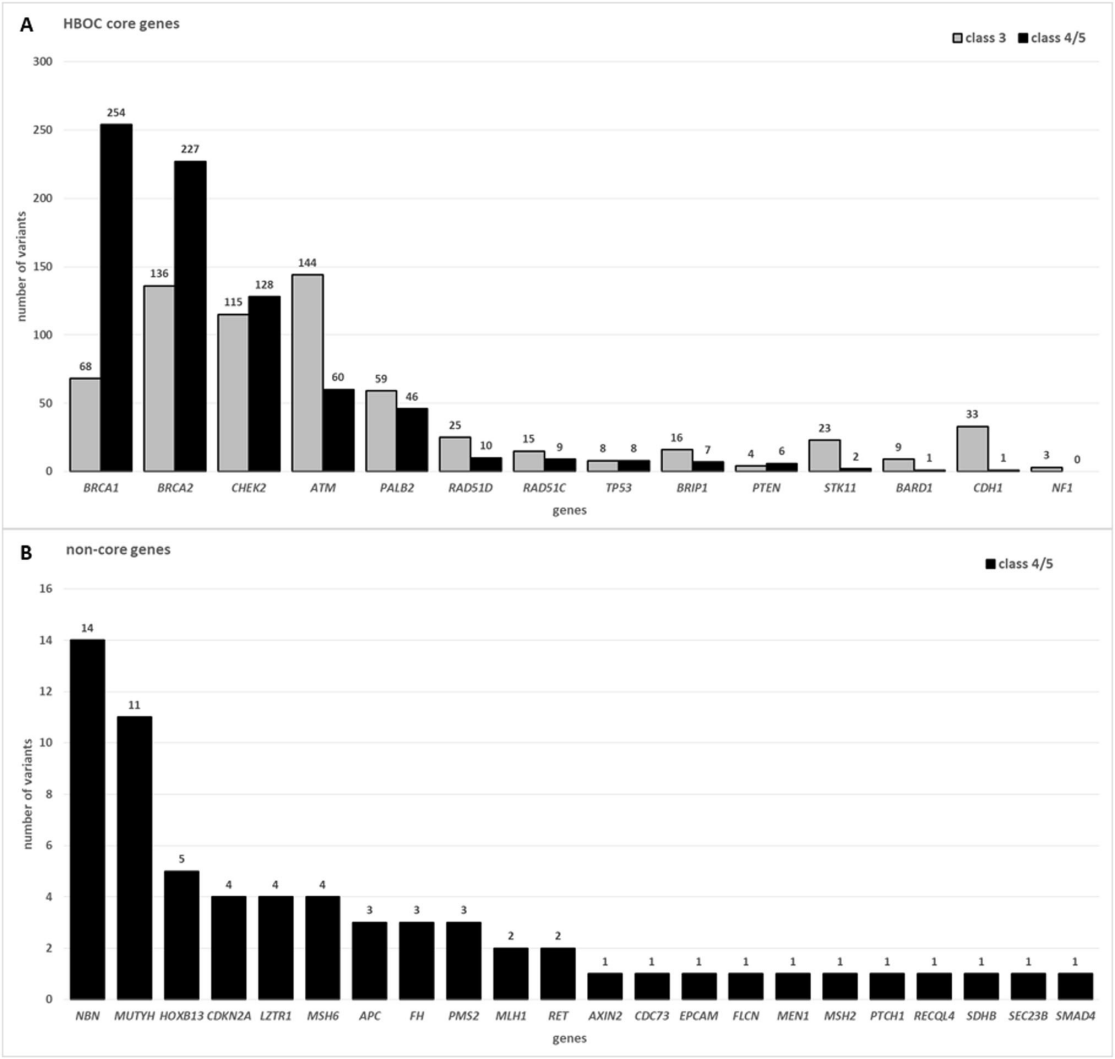
Distribution of reported variants across 123 cancer-associated genes. **A** Descending distribution based on the number of pathogenic (ACMG/AMP class 4/5) variants (*n* = 66) in the HBOC core gene set (*n* = 14). The variants of unknown significance are depicted as grey bars. The numbers on top of the bars, correspond to the number of variants reported in the corresponding gene. **B** Reported pathogenic (ACMG/AMP class 4/5) variants outside the HBOC core gene set associated with other TRS than HBOC. Only genes with reported pathogenic (ACMG/AMP class 4/5) variants are shown (Supplementary Table S2).

A total of 1417 ACMG/AMP class 3 to 5 variants were reported in our defined HBOC core panel representing 92.1% of the 1538 overall identified variants by the comprehensive panel (Supplementary Table S1). The pathogenic variants within the HBOC core panel (ACMG/AMP class 4 and 5) were distributed according to Fig. 1A, with *BRCA1* (*n* = 254), *BRCA2* (*n* = 227), *CHEK2* (*n* = 128), *ATM* (*n* = 60) and *PALB2* (*n* = 46) being the top 5 genes. The distribution of VUS within our HBOC core panel followed a similar descending frequency pattern with *ATM* (*n* = 144) as the top gene followed by *BRCA2* (*n* = 136), *CHEK2* (*n* = 115), *BRCA1* (*n* = 68) and *PALB2* (*n* = 59), completing the top 5 genes (Fig. 1A).

The by the HBOC core panel missed 121 variants (7.9%) were located in thereafter named non-core genes as shown in Supplementary Table S2. Of these 121 non-core gene variants, 66 are reported as class 4/5 and 55 VUS. The majority of VUS are located within *NBN* (*n* = 16), *MSH6* (*n* = 10) and *MLH1* (*n* = 7) (Supplementary Table S2). The pathogenic variants outside the HBOC core panel (*n* = 66) resulting in the diagnosis of other TRS than HBOC as shown in Fig. 1B.

**Fig. 2:**
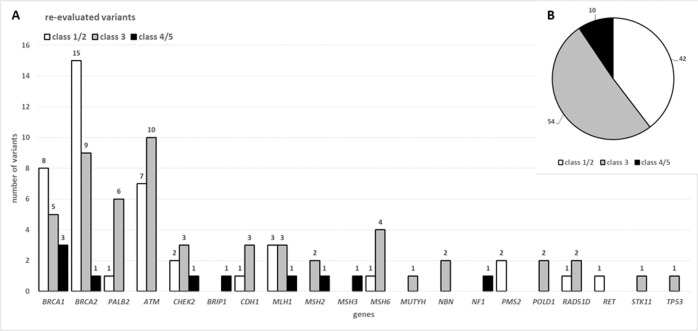
Results of periodic re-evaluated variants of uncertain significance. **A** Displayed are the first 106 re-evaluated VUS associated with different TRS. Grey bars represent VUS, which stayed uncertain, white bars show VUS re-classified as (likely) benign and black bars show VUS re-classified as (likely) pathogenic. The classification is based on the ACMG/AMP guidelines. **B** A pie chart showing the re-evaluated VUS grouped in the different fractions. The grey fraction represent the 51% (*n* = 54) VUS remaining uncertain, the white fraction shows the 40% (*n* = 42) VUS re-classified as (likely) benign and the black fraction represent the 9% (*n* = 10) VUS re-classified as (likely) pathogenic.

Of 106 VUS re-evaluated so far, 9% (*n* = 10) could be re-classified as pathogenic (ACMG/AMP class 4 or 5), 40% (*n* = 42) could be re-classified as benign (class 1 or 2) and 51% (*n* = 54) remained uncertain (ACMG/AMP class 3) (Fig. 2 and Supplementary Table S3).

## Discussion

Although there are recommendations at national and international level as to which genes should be analysed in the context of a hereditary breast and ovarian cancer diagnosis, the individual composition of the corresponding gene panels varies greatly, inevitably resulting in differences regarding the diagnostic yield. For patients who fulfil the German criteria for HBOC testing (AGO guideline), the German HBOC Consortium recommended a panel comprising 10 genes (*ATM*, *BRCA1*, *BRCA2*, *BRIP1*, *CDH1*, *CHEK2*, *PALB2*, *RAD51C*, *RAD51D* and *TP53*) which has only recently been upgraded to 11 genes (*BARD1* added) [19]. The ClinGen “Breast/Ovarian Cancer Gene Curation Expert Panel” (https://www.clinicalgenome.org/affiliation/40042/) suggests analysing 11 genes each for BC and for OC in the diagnostic setting, whereby only the genes *BRCA1*, *BRCA2* and *PALB2* are included in both sets, the genes *ATM*, *CDH1*, *BARD1*, *CHEK2*, *PTEN*, *TP53*, *STK11* and *RECQL* are only recommended for BC and the genes *MLH1*, *MSH2*, *MSH6*, *PMS2*, *EPCAM*, *RAD51C*, *RAD51D* and *BRIP1* are only included in the OC setting [16]. Consulting the curated gene panel lists from the Genomics England PanelApp (https://panelapp.genomicsengland.co.uk/), 5 genes are recommended for the “Inherited breast cancer and ovarian cancer” panel (*ATM*, *BRCA1*, *BRCA2*, *CHEK2* and *PALB2*), the “Pertinent cancer susceptibility” panel also suggests 5 genes only partially overlapping with the first panel (*BRCA1*, *BRCA2*, *PALB2*, *TP53* and *PTEN*) and another panel “Familial breast cancer” contains a fairly comprehensive list of 26 genes (https://panelapp.genomicsengland.co.uk/panels/158/). Others have demonstrated that the application of an expanded gene panel for hereditary cancer testing significantly increased the diagnostic yield compared to guideline-based targeted testing [25, 27].

Since there is no consensus on a single gene panel for HBOC and the German reimbursement system obligatorily requires the analysis of only the two main BC genes *BRCA1* and *BRCA2*, we agreed on a 14-gene core panel for the HBOC diagnostic report (containing the 11 gene panel of the German consortium plus *PTEN*, *STK11* and *NF1*; Fig. 1) with the option, subject to patient consent, to evaluate the other tumour-associated genes of the 123–gene “Hereditary Cancer Syndromes—Comprehensive Panel”.

We are aware that the genes *PTEN* (MIM# 158350, Cowden syndrome), *STK11* (MIM# 175200, Peutz-Jeghers syndrome) and *NF1* (MIM# #162200, Neurofibromatosis type 1) are associated with syndromes that are not primarily characterised by breast and ovarian cancer. As BC and/or OC are an important part of the clinical spectrum of these syndromes, we have decided to integrate both genes into the 14-gene HBOC core panel.

Analysing only the 5 genes from the two different PanelApp BC Panels (*n* = 800 and 1184 cases) would miss a diagnosis in 11.1% (*n* = 148) or 39.9% (*n* = 532) of cases in comparison to our 14-gene core panel (*n* = 1332 cases, Supplementary Table S1). Even if the latest 11 gene panel of the German HBOC consortium (*n* = 1302 cases) were taken as a basis, a diagnosis would be missed in 2.5% (*n* = 30 cases, Supplementary Table S1). In the present study 10.8% of patients had a pathogenic variant (ACMG/AMP class 4/5) in one of the genes of our HBOC core panel (19.2% overall diagnostic yield, Supplementary Table S1), which is in line with the results published elsewhere [28] and demonstrates, that our approach has a high diagnostic yield with a comparatively small panel size.

Our results are consistent with previous observations that a significant proportion of patients with hereditary cancer predisposition were not detected by the guideline-based gene panels used at the time of diagnosis [25, 27–30] and also suggest that expanded diagnostics compared to current multi-gene panels may identify additional patients at high risk for developing other cancers. These findings would allow opportunities for surveillance and, in a small subset of cases, risk reducing measures for patients and their family members who would not have been detected with the guidelines-based gene panels currently in use.

As shown in Fig. 1B, most pathogenic variants outside the 14-core gene panel were found in the genes *NBN* and *MUTYH*, respectively. However, since both genes are associated with recessive tumour predisposition syndromes, the frequency of pathogenic variants in the unselected population may be high, especially for common (founder) mutations in these genes. Indeed, the three most common pathogenic variants in *NBN* (NM_002485.5:c.657_661del p.(Lys219fs) *n* = 9) and *MUTYH* (NM_001048174.2:c.452A > G p.(Tyr151Cys) *n* = 2, NM_001048174.2:c.1103G > A p.(Gly368Asp) *n* = 6) were responsible for 9/14 and 8/11 of pathogenic findings (Fig. 1B and Supplementary Table S2). For monoallelic carriers of pathogenic variants in *NBN* and *MUTYH*, however, there is no increase in risk in the sense of penetrant tumour disease and thus no clinical actionability. For many of the other genes on the secondary findings list (Fig. 1B), knowledge about a predisposition for a TRS is extremely important, as targeted surveillance examinations can be initiated in the index patient and, if necessary, family members with an increased risk can be identified and included in surveillance programmes. In some of the secondarily identified TRS, BC can be part of the clinical spectrum and at least for the Lynch-syndrome cases would have therapeutic consequences.

For this reason, we suggest not to use rigid gene panels, which by definition are not complete and at the same time often not specific enough, but to start with a core BC-OC panel (ideally containing all HBOC associated genes [16, 17]) and taking into account the individual cancer history and family history and in case of a negative result to extend the analysis to other cancer-associated genes. The initial introduction of core panels for HBOC diagnostics in Germany is based on the recommendations of the German Breast Cancer Consortium in order to meet the requirements of the German reimbursement system (only the genes *BRCA1* and *BRCA2* are obligatory; other HBOC associated genes can be optionally reported). Nevertheless, we appreciate the issues which accompany the universal application of expanded gene panels, namely the uncertain clinical utility of identifying ACMG/AMP class 4/5 variants in genes outside the recommended ones based on established guidelines and the potential burden of variants of uncertain significance (VUS) [12, 31]. This burden or fear is used by some as an argument to use only small guideline-based gene panels for diagnostic tests [32]. Yet, we and others believe that a possible psychological burden may well exist in some individuals who have an “incidental” or “additional” finding reported [33, 34]. However, we would like to state that it is more meaningful to treat this anxiety professionally in these affected individuals rather than denying all patients the irrefutable benefit of additional findings of actionable pathogenic variants. In the first place, according to the German Genetic Diagnostics law and national recommendations, secondary/incidental findings are only reported if the patient has received genetic counselling prior to the test and has explicitly given his or her consent; this consent can be revoked up to the time of reporting the findings. In general, there is a wide range of psychological counselling available in Germany and we even have our own in-house psycho-oncological counselling centre for our patients.

As others have demonstrated [20–24], regular reassessment of VUS can dramatically improve the clinical validity of genetic reports, e.g. half of all VUS can be reclassified as (likely) benign and about 10% as (likely) pathogenic (Fig. 2) [13, 14]. These results are based on a re-evaluation using new data (population data, functional data, segregation data, etc.) with the help of the current SVI recommendations on the use of the ACMG/AMP classification guidelines. Especially for the hereditary tumour syndromes (HBOC and CRC), special adaptations of the original ACMG/AMP codes are currently being created by so-called Variant Curation Expert Panels (VCEP’s). We therefore expect a significantly improved data situation in the next few years, as these VCEPs will reclassify many discordant variants and VUS in the ClinVar database on the basis of these new codes and provide guidance for how to improve variant classification (e.g. by defining thresholds for when a variant is too frequent for a disease, what frequency can be used to filter for “rare enough” variants, which regions could be regarded as mutational hot-spots and what functional studies should be used, etc.). A controlled, regular re-evaluation of VUS in diagnostic laboratories is therefore imperative, as valuable clinical information can be collected that can relieve patients and their family members as well as improve care for carriers of pathogenic variants.

In this context, it is important to note that the main reason for discordant variant classifications in ClinVar or LOVD is the lack of data sharing from diagnostic laboratories [35, 36]. Data sharing is the pivotal prerequisite for further improving UV classification in a timely manner and would therefore be of great benefit for many patients and their families.

To conclude, we suggest to use the outlined strategy to start with a core panel followed by a comprehensive panel as this strategy can identify additional TRS in patients referred to HBOC testing, which would not have been detected by current rigid guideline-based testing. Knowledge about a predisposition for a TRS is extremely important, as targeted surveillance examinations can be initiated in the index patient and, if necessary, family members with an increased risk can be identified and included in surveillance programmes.

## Supplementary information

Table S1

Table S2

Table S3

## Data Availability

The datasets generated and/or analysed during the current study are available from the corresponding author on reasonable request.

## References

[1] Siegel R, Naishadham D, Jemal A. Cancer statistics, 2013. CA Cancer J Clin. 2013;63:11–30. 10.3322/caac.21166 23335087

[2] Eccles SA, Aboagye EO, Ali S, Anderson AS, Armes J, Berditchevski F (2013). Critical research gaps and translational priorities for the successful prevention and treatment of breast cancer. Breast Cancer Res.

[3] Easton DF, Pharoah PD, Antoniou AC, Tischkowitz M, Tavtigian SV, Nathanson KL (2015). Gene-panel sequencing and the prediction of breast-cancer risk. N Engl J Med.

[4] Crawford B, Adams SB, Sittler T, van den Akker J, Chan S, Leitner O (2017). Multi-gene panel testing for hereditary cancer predisposition in unsolved high-risk breast and ovarian cancer patients. Breast Cancer Res Treat.

[5] Mavaddat N, Michailidou K, Dennis J, Lush M, Fachal L, Lee A (2019). Polygenic risk scores for prediction of breast cancer and breast cancer subtypes. Am J Hum Genet.

[6] Chandler MR, Bilgili EP, Merner ND (2016). A review of whole-exome sequencing efforts toward hereditary breast cancer susceptibility gene discovery. Hum Mutat.

[7] Turnbull C, Rahman N (2008). Genetic predisposition to breast cancer: past, present, and future. Annu Rev Genomics Hum Genet.

[8] Chenevix-Trench G, Healey S, Lakhani S, Waring P, Cummings M, Brinkworth R (2006). Genetic and histopathologic evaluation of BRCA1 and BRCA2 DNA sequence variants of unknown clinical significance. Cancer Res.

[9] Eggington JM, Bowles KR, Moyes K, Manley S, Esterling L, Sizemore S (2014). A comprehensive laboratory-based program for classification of variants of uncertain significance in hereditary cancer genes. Clin Genet.

[10] Lindor NM, Guidugli L, Wang X, Vallée MP, Monteiro AN, Tavtigian S (2012). A review of a multifactorial probability-based model for classification of BRCA1 and BRCA2 variants of uncertain significance (VUS). Hum Mutat.

[11] Tutt A, Robson M, Garber JE, Domchek SM, Audeh MW, Weitzel JN (2010). Oral poly(ADP-ribose) polymerase inhibitor olaparib in patients with BRCA1 or BRCA2 mutations and advanced breast cancer: a proof-of-concept trial. Lancet..

[12] Eccles DM, Mitchell G, Monteiro AN, Schmutzler R, Couch FJ, Spurdle AB (2015). BRCA1 and BRCA2 genetic testing-pitfalls and recommendations for managing variants of uncertain clinical significance. Ann Oncol.

[13] Kwong A, Ho CYS, Shin VY, Au CH, Chan TL, Ma ESK (2022). How does re-classification of variants of unknown significance (VUS) impact the management of patients at risk for hereditary breast cancer?. BMC Med Genomics.

[14] Mighton C, Shickh S, Uleryk E, Pechlivanoglou P, Bombard Y (2021). Clinical and psychological outcomes of receiving a variant of uncertain significance from multigene panel testing or genomic sequencing: a systematic review and meta-analysis. Genet Med.

[15] Richards S, Aziz N, Bale S, Bick D, Das S, Gastier-Foster J (2015). Standards and guidelines for the interpretation of sequence variants: a joint consensus recommendation of the American College of Medical Genetics and Genomics and the Association for Molecular Pathology. Genet Med.

[16] Lee K, Seifert BA, Shimelis H, Ghosh R, Crowley SB, Carter NJ (2019). Clinical validity assessment of genes frequently tested on hereditary breast and ovarian cancer susceptibility sequencing panels. Genet Med.

[17] Dorling L, Carvalho S, Allen J, González-Neira A, Luccarini C, Breast Cancer Association Consortium (2021). Breast cancer risk genes—association analysis in more than 113,000 women. N Engl J Med.

[18] Desmond A, Kurian AW, Gabree M, Mills MA, Anderson MJ, Kobayashi Y (2015). Clinical actionability of multigene panel testing for hereditary breast and ovarian cancer risk assessment. JAMA Oncol.

[19] Wappenschmidt B, Hauke J, Faust U, Niederacher D, Wiesmüller L, Schmidt G (2020). Criteria of the German Consortium for Hereditary Breast and Ovarian Cancer for the Classification of Germline Sequence Variants in Risk Genes for Hereditary Breast and Ovarian Cancer. Geburtshilfe Frauenheilkd.

[20] So MK, Jeong TD, Lim W, Moon BI, Paik NS, Kim SC (2019). Reinterpretation of BRCA1 and BRCA2 variants of uncertain significance in patients with hereditary breast/ovarian cancer using the ACMG/AMP 2015 guidelines. Breast Cancer.

[21] Bennett JS, Bernhardt M, McBride KL, Reshmi SC, Zmuda E, Kertesz NJ (2019). Reclassification of variants of uncertain significance in children with inherited arrhythmia syndromes is predicted by clinical factors. Pediatr Cardiol.

[22] SoRelle JA, Thodeson DM, Arnold S, Gotway G, Park JY (2019). Clinical utility of reinterpreting previously reported genomic epilepsy test results for pediatric patients. JAMA Pediatr.

[23] Slavin TP, Manjarrez S, Pritchard CC, Gray S, Weitzel JN (2019). The effects of genomic germline variant reclassification on clinical cancer care. Oncotarget..

[24] Chiang J, Chia TH, Yuen J, Shaw T, Li ST, Binte Ishak ND (2021). Impact of variant reclassification in cancer predisposition genes on clinical care. JCO Precis Oncol.

[25] Stenson PD, Mort M, Ball EV, Evans K, Hayden M, Heywood S (2017). The Human Gene Mutation Database: towards a comprehensive repository of inherited mutation data for medical research, genetic diagnosis and next-generation sequencing studies. Hum Genet.

[26] Landrum MJ, Lee JM, Benson M, Brown G, Chao C, Chitipiralla S (2016). ClinVar: public archive of interpretations of clinically relevant variants. Nucleic Acids Res.

[27] Ceyhan-Birsoy O, Jayakumaran G, Kemel Y, Misyura M, Aypar U, Jairam S (2022). Diagnostic yield and clinical relevance of expanded genetic testing for cancer patients. Genome Med.

[28] Beitsch PD, Whitworth PW, Hughes K, Patel R, Rosen B, Compagnoni G (2019). Underdiagnosis of hereditary breast cancer: are genetic testing guidelines a tool or an obstacle?. J Clin Oncol.

[29] Samadder NJ, Riegert-Johnson D, Boardman L, Rhodes D, Wick M, Okuno S (2021). Comparison of universal genetic testing vs guideline-directed targeted testing for patients with hereditary cancer syndrome. JAMA Oncol.

[30] Mandelker D, Zhang L, Kemel Y, Stadler ZK, Joseph V, Zehir A (2017). Mutation detection in patients with advanced cancer by universal sequencing of cancer-related genes in tumor and normal DNA vs guideline-based germline testing. JAMA..

[31] van der Post RS, Vogelaar IP, Carneiro F, Guilford P, Huntsman D, Hoogerbrugge N (2015). Hereditary diffuse gastric cancer: updated clinical guidelines with an emphasis on germline CDH1 mutation carriers. J Med Genet.

[32] de Wert G, Dondorp W, Clarke A, Dequeker EMC, Cordier C, Deans Z (2021). Recommendations of the European Society of Human Genetics. Eur J Hum Genet.

[33] Biesecker LG (2022). Invited Commentary on “My Research Results: a program to facilitate return of clinically actionable genomic research findings” by Willis et al. Eur J Hum Genet.

[34] Parens E, Appelbaum PS (2019). On what we have learned and still need to learn about the psychosocial impacts of genetic testing. Hastings Cent Rep.

[35] Amendola LM, Jarvik GP, Leo MC, McLaughlin HM, Akkari Y, Amaral MD (2016). Performance of ACMG-AMP variant-interpretation guidelines among nine laboratories in the Clinical Sequencing Exploratory Research Consortium. Am J Hum Genet.

[36] Harrison SM, Dolinsky JS, Knight Johnson AE, Pesaran T, Azzariti DR, Bale S (2017). Clinical laboratories collaborate to resolve differences in variant interpretations submitted to ClinVar. Genet Med.

